# Neuroendocrine breast carcinoma: a rare but challenging entity

**DOI:** 10.1007/s12032-020-01396-4

**Published:** 2020-07-25

**Authors:** Elena Trevisi, Anna La Salvia, Lorenzo Daniele, Maria Pia Brizzi, Giovanni De Rosa, Giorgio V. Scagliotti, Massimo Di Maio

**Affiliations:** 1 Department of Oncology, University of Turin, San Luigi Gonzaga Hospital, Regione Gonzole 10, 10043 Orbassano, Turin Italy; 2 Department of Oncology, University Hospital, 12 de Octubre, Madrid, Spain; 3 Pathology Unit, Ordine Mauriziano Hospital, Torino, Italy; 4 Department of Oncology, University of Turin, Ordine Mauriziano Hospital, Torino, Italy

**Keywords:** Breast cancer, Neuroendocrine breast carcinoma, Neuroendocrine differentiation, Small cell breast cancer, Neuroendocrine tumor

## Abstract

Breast carcinoma with neuroendocrine differentiation, also known as neuroendocrine breast carcinoma (NEBC), includes a heterogeneous group of rare tumors, which account for 2–5% of all invasive breast carcinomas. Because of their low incidence, most of the current limited knowledge of these tumors derives from anecdotal case reports or small retrospective series. The diagnosis of NEBC is based on the presence of morphological features similar to gastrointestinal and lung NETs and neuroendocrine markers. NEBCs are usually hormone receptors positive and HER2 negative, but despite this luminal phenotype, most recent studies suggested that NEBC could be associated with worse prognosis compared to invasive breast cancer without neuroendocrine differentiation. Due to its rarity and lack of randomized data, there is little evidence to guide the choice of treatment, so NEBC is currently treated as any invasive breast carcinoma not-otherwise specified. Recently, attempts to molecularly characterize NEBC have been made, in order to provide new targets for a more personalized treatment of this uncommon entity.

## Introduction

Breast carcinoma with neuroendocrine differentiation, also known as neuroendocrine breast carcinoma (NEBC), includes a heterogeneous group of rare tumors, which account for 2–5% of all-invasive breast carcinomas [1]. Because of their low incidence, most of the current limited knowledge of these tumors derives from anecdotal case reports or small retrospective series. Their definition, prevalence, and prognosis remain controversial in literature. To date, there is no standard treatment specifically tested in NEBC.

In this review, we summarize the current evidence and the main challenges about epidemiology, histopathological and immunohistochemical features, diagnosis, prognosis, and treatment of NEBC. We also discuss new insights and novel potential therapeutic targets, resulting from a better molecular knowledge of this uncommon entity.

## Methods

On January 2020, we performed a comprehensive literature review of the PubMed database concerning NEBC using terms “breast” AND (“neuroendocrine differentiation” OR “neuroendocrine carcinoma” OR “neuroendocrine tumor”). The search was limited to articles published in English.

### Histopathological and immunohistochemical features

NEBC was first described in 1963 by Feyrter and Hartmann as carcinoid growth pattern within two cases of breast cancer [2]. Later, in 1977, Cubilla and Woodruff classified eight cases of breast cancers as “carcinoid” [3]. Only several years later, in 2003, the World Health Organization (WHO) recognized NEBC as a separate entity of breast cancer, showing morphological characteristics similar to gastrointestinal and pulmonary neuroendocrine tumors (NETs), with the expression of a neuroendocrine marker in at least 50% of tumor cells [4]. Chromogranin A (CgA) and synaptophysin (Syn) are the most sensitive neuroendocrine markers [5–7], whereas neuron-specific enolase (NSE) and CD56 are less sensitive and less specific [8, 9]. In 2012, WHO classification was revised and the threshold value of > 50% of neuroendocrine marker expression in tumor cells was removed, since this cut-off was considered arbitrarily set. According to the 2012 WHO classification, these tumors were categorized into three groups: well differentiated NEBC (NETs, which included low- and intermediate-grade tumors), poorly differentiated NEBC/small cell carcinoma, and NEBC determined by histochemistry or immunohistochemistry (IHC) [1]. The latter category included breast carcinoma of no special type (NST), as well as special type such as solid papillary carcinoma and the hypercellular subtype of mucinous carcinoma. Indeed, as described by Capella et al., the so-called type B of mucinous carcinoma often show neuroendocrine differentiation [10]. According to the 2012 WHO classification, the distinction between NETs and grade 1 or 2 breast carcinomas of other types that show neuroendocrine differentiation was not so clear. For this reason, the key feature of the 2019 WHO classification is the distinction between well-differentiated NETs and poorly differentiated neuroendocrine carcinomas (NECs), and breast neuroendocrine neoplasms are now categorized as NETs, small cell NECs and large cells NECs [11].

**Fig. 1:**
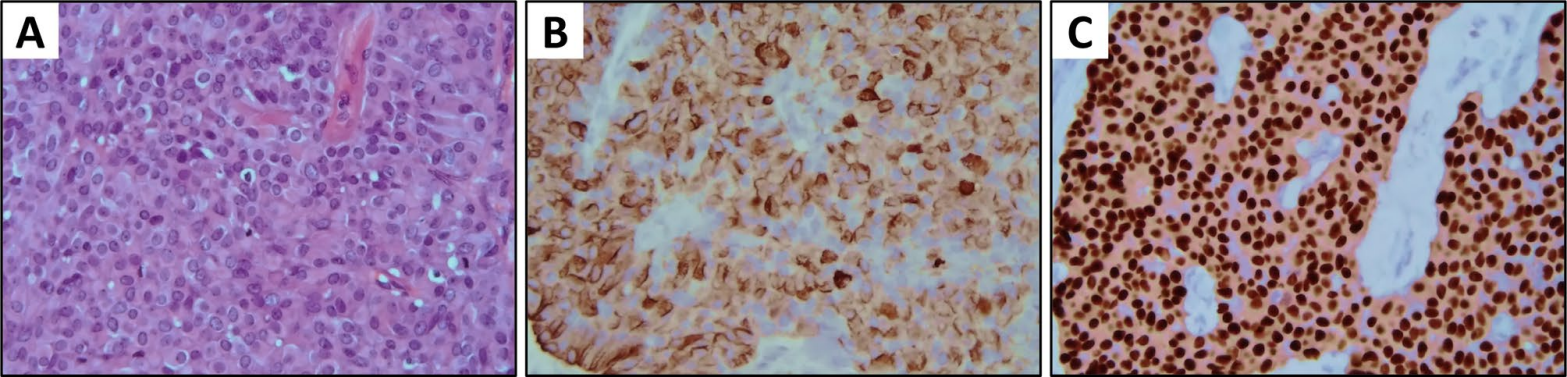
Pathological findings in a large cells neuroendocrine carcinoma of the breast (WHO [11]). **a** H&E stain, ×10, **b** on immunohistochemistry, tumor cells show diffuse positive stain for Chromogranin A (×10), and **c** estrogen receptors (×10)

NEBCs are typically hormone receptors (HR) positive and human epidermal growth factor receptor 2 (HER2) negative [12–14] (Fig. 1). They can belong to either the luminal A or luminal B molecular subtypes. In 2008, Weigelt et al. described a limited cohort of 6 NEBCs, with 5 cases of luminal A tumor and 1 case of luminal B tumor [12]. In a larger series, Bogine et al. subdivided 112 NEBCs with luminal phenotype as 42% luminal A and 58% luminal B [13]. Similarly, in their series of 47 NEBCs, Lavigne et al. reported 52% of their cases as luminal A and 48% as luminal B [14]. On the other hand, HER2 is only sporadically amplified [15–17]. Somatostatin receptors (SSTRs) are G-protein coupled receptors expressed by NET cells at lung, prostate and gastrointestinal level, as well as by ductal breast cancer cells. There are five known subtypes of SSTRs (named SSTR1 to SSTR5), with SSTR2A being the subtype most commonly expressed in breast cancer and being most closely associated with luminal tumors [18–20]. Recently, the presence of SSTR2A and SSTR5 has been investigated in NEBCs. Namely, in a retrospective series of 31 cases, the total percentage showing a positive membrane IHC reaction was 71% for both SSTR2A and SSTR5 [21].

### Epidemiology

NEBC is a rare entity. The reported prevalence of NEBC among breast cancers varies from 0.1 to 18% [1, 4, 22–24]. According to the 2003 classification, the prevalence of these tumors was estimated between 2 and 5%. However, in the Surveillance Epidemiology and End Results (SEER) database, only 142 cases of NEBC were identified during the period from 2003 to 2009, which corresponded to a prevalence of < 0.1% [24]. The lack of uniform morphological and immunohistochemical diagnostic criteria may explain the different prevalence reported in the literature.

Similarly to the more frequent types of breast cancer, NEBC is more common in female patients between the sixth and seventh decade of age [24]. However, few cases have been diagnosed even in the premenopausal period [15, 25] or in male patients [26–28].

### Prognosis

The prognostic implications of neuroendocrine differentiation in breast carcinoma remain controversial. Historically, based on small studies, NEBC was thought to have prognosis similar [22, 23, 29], or even better [30, 31], compared to invasive ductal carcinoma of no special type. However, most recent studies suggested that NEBC could be associated with worse long-term outcomes [24, 32–36]. Among these, the population-based study from SEER database showed that overall survival (OS) and disease-specific survival (DSS) were significantly shorter in NEBC compared with non-NEBC at the same stage [24]. Also a large retrospective study conducted by Zhang et al. showed a higher probability of local recurrence and poorer OS for NEBCs [34]. Of course, the limited number of studies reported in literature and the lack of uniformity in the definition and classification may affect these conflicting results concerning the clinical outcome of NEB. Likewise, some authors investigated the possible impact of histologic subtyping of NEBC according to the 2012 WHO classification on prognosis, providing different evidences. Cloyd et al. showed that small cell carcinoma subtype is associated with worse DSS and OS compared to well-differentiated NECB and invasive carcinoma with neuroendocrine features [36]. More recently, in a small series of 47 patients, Lavigne et al. did not find any statistically significant difference in terms of prognosis between the three subtypes, although OS and progression-free survival (PFS) of the seven poorly differentiated neuroendocrine carcinomas were actually worse compared with the other two groups [14].

### Diagnosis

The diagnosis of NECB can be challenging. NEBCs have no distinctive presenting signs or symptoms. Very rarely, NEBC can present peculiar clinical features related to hormonal hypersecretion [37]. In fact, anecdotal cases of patients showing symptoms secondary to ectopic secretion of calcitonin, norepinephrine or ACTH have been described in the literature [38–40]. Similarly to typical luminal subtypes of breast cancer, NEBC can metastasize to several sites, but more frequently to bone and liver [37, 41].

The imaging findings in patients with NEBC are not specific, and comparable to the ones of other types of breast tumors. Some authors showed that NEBCs may present on mammography as well circumscribed lesions, with no associated microcalcifications, and on ultrasonography, as hypoechogenic mass with irregular morphology and ill-defined margins, with or without cystic component. On magnetic resonance, NECB was described—at least in some cases—as a hypointense irregular lesion on T1-weighted sequences, with early and intense enhancement [42–44]. 

Since the diagnosis of NEBC is based on morphological features and neuroendocrine markers, a biopsy is required for definitive diagnosis. Because of their rarity as primary breast carcinomas, metastasis from another primary neuroendocrine tumor should be always excluded for a differential diagnosis. The presence of a ductal carcinoma in situ component is consistent with the primary nature of the tumor [45].

To exclude a different primary site, a chest and abdomen computed tomography (CT) scan should be performed. Moreover, for the same purpose, a positron emission tomography (PET)-CT with 68Gallium-labeled somatostatin analogs can be performed in the case of well-differentiated NEBC, whereas 18-fluorodeoxyglucose PET-CT could be useful in the case of poorly differentiated NEBC or small carcinomas with a high proliferation rate [46, 47].

### Treatment

Due to its rarity and lack of randomized data, there is little evidence to guide the choice of treatment. Consequently, NEBC is currently treated as any invasive breast carcinoma not-otherwise specified.

Surgery is the mainstay of the treatment for early NECB, and the choice of surgical procedure depends on the location of the tumor and on the clinical stage. Since there are no robust data on the role of adjuvant/neoadjuvant therapy in NEBC, it should be prudentially considered according to the same indications adopted for the other types of invasive breast cancer. Likewise, there are no NEBC-specific treatments in the metastatic setting. As for the more common types of breast cancer, treatment strategy should be based on the tumor burden and biological features, as well as the age, menopausal status, general conditions and preferences of the patient [48].

As described above, NEBCs usually exhibit a luminal phenotype; considering that endocrine therapy (ET) has a well-established role in the treatment of HR-positive breast cancer, therefore, it could be a useful tool in the management of NEBCs [49–51]. The addition of a cyclin-dependent kinase (CDK) 4/6 inhibitor to an aromatase inhibitor has significantly changed the prognosis of metastatic patients, both naïve and pre-exposed to ET, providing a great benefit in terms of PFS and, at least in some studies, in terms of OS [52–54]. Namely, the combination of palbociclib and fulvestrant has been used in the treatment of a patient affected from NEBC, with positive results [55]. The patient, affected by high-grade NEBC, was refractory to platinum-based chemotherapy as well as first-line hormonal treatment with tamoxifen and leuprolide, but showed a durable response to fulvestrant plus palbociclib.

Everolimus has demonstrated efficacy in well differentiated pancreatic, gastrointestinal and lung NETs [56–58]. Further` more, this drug has a specific indication in breast cancer patients, because, in addition to exemestane, it significantly prolonged PFS in metastastic HR positive, HER2-negative breast cancer previously exposed to ET [59]. In light of both these data, a combination of everolimus and exemestane could be an effective treatment option in patients with metastatic luminal well differentiated NEBC.

Anti-HER2 therapy can be used in sporadic cases of NEBC with HER2 overexpression [17].

To date, there is no evidence to select the most effective chemotherapy regimen. The choice of chemotherapy agents can be based on the histopathological features of NEBC. Usually, poorly differentiated or small cell NEBCs have been treated with platinum/etoposide-containing regimes, that is the standard treatment for small cell lung cancer and similar high-grade poorly differentiated neuroendocrine tumors, while anthracyclines and/or taxanes-based chemotherapy have been used for other types of NEBCs [60, 61].

SSTRs are targets for biological therapy in NETs. Somatostatin analogs (SSA) showed an antiproliferative role, providing a prolongation of PFS in small intestinal NETs and they are recommended by international guidelines for the first-line therapy of well-differentiated, G1/2 metastatic NETs [62, 63]. Peptide receptor radionuclide therapy (PRRT), which consists of a radiolabeled somatostatin analogue, is a novel emerging treatment option for patients with well-differentiated metastatic NETs expressing SSTRs [64, 65]. Similarly to NETs from other sites, SSTRs in NEBC could be a potential therapeutic target [66, 67].

### New molecular insights

In the last years, several efforts have been made to identify potential targets for novel therapeutic approaches in NEBC.

In 2014, a first molecular characterization was provided by Ang et al., who found a PIK3CA mutation in 20% of NEBCs [68]. It seems that NEBCs harbour PIK3CA mutations in a variable percentage, ranging between 7 and 33% [14, 68–70], less frequently than common HR positive, HER2 negative breast cancer (up to 45%) [71, 72]. Nevertheless, targeting PIK3CA in metastatic NECB could represent an intriguing therapeutic strategy, given the recent results achieved with the use of alpelisib in a population affected from HR positive, HER2 negative advanced breast cancer [73]. In their recent series, Vranic et al. found a TROP-2 protein expression in 21%, suggesting that a small proportion of NEBCs may be sensitive to target therapy with sacituzumab govitecan [74, 75]. To date, all currently approved biomarkers of response to immune checkpoint inhibitors (PD-L1 expression, high tumor mutational burden and microsatellite instability status) have proven negative, suggesting that patients with NEBC are not ideal candidates for immunotherapy [69, 74] (Table 1).

**Table 1:** An overview of pathological and clinical features of NEBC case reports published over the past 10 years

Author (year)	Age	T size(mm)	Staging	ER	PR	HER2	Ki67 (%)	Surgery	ET	Chemo	F-U (mo)	Status
Nicoletti (2010) [76]	40	30	IIB	+	+	−	90	M + Ax	Yes	AC + CBDCA/VP-16	96	NED
Christie (2010) [77]	61	45	IIIC	−	−	−	NS	WLE + Ax	No	CBDCA/VP-16	3	DOD
Latif (2010) [78]	53	50	IIB	−	−	−	NS	WLE	No	CBDCA/VP-16	~ 6	NED
Nozoe (2011) [79]	57	30	IIA	+	+	−	NS	M + Ax	Yes	FEC + DTX	NS	NED
Buttar (2011) [49]	63	/	IIA	+	+	−	NS	M	Yes	No	48	AWD
Honami (2011) [80]	54	10	IA	+	+	−	NS	WLE	Yes	No	18	NED
Zhang (2011) [81]	29	85	IIB	+	+	−	< 1	WLE	Yes	FEC + DTX	20	NED
Yildirim (2011) [82]	70	45	IIB	+	+	−	< 10	M	Yes	No	37	NED
	30	35	IIB	−	−	−	60	M	No	CDDP/VP-16	35	NED
	74	40	IIA	+	+	−	< 10	M	No	No	46	NED
	40	45	IIIA	+	+	−	10	M	Yes	FEC	52	NED
	75	40	IIA	+	+	−	< 10	M	Yes	No	13	NED
	35	20	IIB	+	+	−	50	M	Yes	CCDP/VP-16	12	AWD
Watrowski (2012) [83]	56	17	IA	+	+	−	46	WLE	Yes	FEC	15	NED
Su (2012) [84]	75	40	IIA	+	+	−	NS	M + Ax	Yes	No	20	NED
Alkaied (2012) [50]	83	/	IV	+	+	−	NS	/	Yes	No	12	AWD
Menéndez (2012) [85]	44	20	IB	+	+	−	NS	WLE + Ax	No	FEC	48	NED
	68	35	NS	NS	NS	NS	NS	WLE + Ax	Yes	FEC	24	NED
	58	10	IA	+	−	−	NS	WLE	No	FEC	8	AWD
	69	15	IA	+	+	−	10	WLE	NS	NS	2	NED
Yavas (2012) [16]	77	45	IIIA	+	+	+	NS	M + Ax	No	No	15	NED
Psoma (2012) [86]	46	65	NS	NS	NS	NS	NS	M + Ax	NS	CDDP/VP-16/EPI	6	NED
Angarita (2013) [60]	51	30	IIIB	+	−	−	> 20	M	Yes	CDDP/VP-16 → CBDCA/PTX	13	AWD
Hanna (2013) [87]	60	15	IIIA	+	+	−	NS	WLE + Ax	No	CBDCA/VP-16	NS	NS
Tajima (2013) [88]	78	15	IIIA	+	−	−	32	M + Ax	Yes	No	12	NED
Jiang (2014) [28]	79	15	IIA	−	+	+	NS	M + Ax	No	CBDCA/CPT-11 → DTX	27	DOD
Pagano (2014) [51]	51	35	IIIA	+	+	−	30	M + Ax	Yes	CMF	240	NED
Manes (2014) [41]	51	NS	IIA	+	−	−	NS	WLE + Ax	Yes	FEC	114	NED
Yoon (2014) [44]	44	22	IIA	+	+	−	NS	WLE	NS	AC	2	NED
Bozkurt (2014) [89]	75	30	IIA	+	+	−	5	M	No	Yes	NS	NS
Adams (2014) [90]	67	9	IA	+	−	−	NS	WLE + Ax	No	No	6	NED
Wei (2015) [61]	43	80	IIIA	+	−	−	40	M	No	EC + DTX	NS	NS
Sherwell-Cabello (2015) [91]	60	60	IIIC	−	−	−	70	M	No	CBDCA/VP-16	6	NED
Yoshimura 2015) [23]	34	60	IIIA	+	+	−	25	M + Ax	No	No	48	NED
Janosky (2015) [92]	34	40	IIA	−	−	−	100	M	No	AC + DTX → CBDCA/PTX → CDDP/VP-16 → ERI	~ 12	AWD
Gevorgyan (2016) [17]	65	NS	IA	+	−	+	5	M + Ax	No	PTX + TRA	379	AWD
Collado-Mesa (2017) [93]	58	10	IA	+	+	−	NS	WLE	NS	NS	NS	NS
	62	14	IA	+	+	−	NS	WLE	NS	NS	NS	NS
Soe (2017) [94]	57	40	IV	+	+	−	15	No	No	CDDP/VP-16	18	AWD
Tremelling (2017) [95]	65	50	IIIA	−	−	−	NS	No	No	CBDCA/VP-16	3	AWD
Abou Dalle (2017) [96]	47	30	IIA	−	+	−	50	M	No	CDDP/VP-16 + FEC	10	NED
Bergstrom (2017) [97]	53	80	NS	+	−	−	90	No	No	CDDP/VP-16	NS	NS
Shanks (2018) [55]	44	16	IV	+	+	−	80	No	Yes	CBDCA/VP-16 → PALB	~ 25	AWD
Valente (2019) [98]	69	25	IIB	+	+	−	90	WLE + Ax	Yes	FEC	96	AWD
Kawasaki (2019) [99]	53	10	IIIA	−	−	−	75	M + Ax	No	EC	5	NED

*ER* estrogen receptor; *PR* progesterone receptor; *HER2* human epidermal growth factor receptor 2; *ET* endocrine therapy; *Chemo* chemotherapy; *F*-*U* (*mo*) follow-up (months); *SURGERY: M* mastectomy; *Ax* axillary dissection; *WLE* wide local excision; *CHEMO: AC* Adriamycin (Doxorubicin)/Cyclophosphamide; *CBDCA* Carboplatin; *CDDP* Cisplatin; *CMF* Cyclophosphamide/Methotrexate/Fluorouracil; *CPT-11* Irinotecan; *DTX* Docetaxel; *EC* Epirubicin/Cyclophosphamide; *EPI* Epirubicin; *ERI* Eribulin; *FEC* Fluorouracil/Epirubicin/Cyclophosphamide; *PALB* Palbociclib; *PTX* Paclitaxel; *TRA* Trastuzumab; *VP*-*16* Etoposide; *STATUS: NED* no evidence of disease; *AWD* alive with disease; *DOD* died of disease. Arrows ( →) indicate a change of regimen

## Conclusions

In summary, NEBC includes a group of rare breast carcinomas, that tend to occur in older women. The diagnosis of NEBC is based on the presence of morphological features similar to gastrointestinal and lung NETs, along with the presence of neuroendocrine markers. Due to its rarity and its recent recognition as a separate entity, the current diagnostic and therapeutic protocol is similar to that of general invasive breast carcinomas. Despite its frequent luminal (A or B) phenotype, most recent studies have reported poorer clinical outcomes for NEBC compared with typical breast carcinomas without neuroendocrine differentiation. Therefore, there is a still unmet need to enhance the ability to identify this uncommon entity, as well as to better know its biology for setting up a more tailored treatment.
